# The development of working alliance in early stages of care from the perspective of patients attending a chiropractic teaching clinic

**DOI:** 10.1186/s12998-023-00527-8

**Published:** 2024-03-21

**Authors:** Dima Ivanova, Dave Newell, Jonathan Field, Felicity L. Bishop

**Affiliations:** 1 School of Psychology, University of Southampton, University Road, Southampton, Hampshire, SO17 1BJ UK; 2 AECC University College, Parkwood Campus, Parkwood Road, Bournemouth, Dorset, BH5 2DF UK; 3 Private Practice, Anstey Road, Alton, Hampshire, UK

**Keywords:** Working Alliance, Therapeutic Alliance, Chiropractor-patient relationship, Context, Expectations, Confidence, Expertise, Explanations, Pain, Trust

## Abstract

**Background:**

The clinician-patient relationship has consistently been found to predict treatment success in both physical and mental health settings. This relationship has been operationalised in the literature as “Working Alliance,” which consists of three key components: patient-clinician agreement on the goals of care, agreement on the tasks required to achieve those goals, and the establishment of a strong bond. While research has demonstrated the impact of working alliance in physical health settings, it often measures working alliance early in patients’ care journeys. However, no primary research has investigated how early working alliance develops between patients and chiropractors. Evidence suggests that musculoskeletal practitioners may require further training to feel confident in establishing working alliance. Therefore, this study aims to explore the development of working alliance in the early stages of chiropractic care from the patients’ perspective to inform evidence-based practice.

**Methods:**

Participants for this qualitative study were recruited from a teaching clinic at a specialised healthcare professions training university in the United Kingdom between September 2022 and April 2023. A total of 25 adult patients completed semi-structured interviews during the early stages of their care. The interview transcripts were analysed using Reflexive Thematic Analysis, from a critical realist stance.

**Results:**

The findings highlight that an early working alliance entails the gradual development of patients’ confidence in their decision to seek help from trainee chiropractors to alleviate their symptoms. The four themes describe the impact of the clinical context on patients’ expectations, the trainee chiropractors’ qualities that participants considered important for early working alliance, the role of explanations, and the interplay between pain and early working alliance.

**Conclusions:**

Establishing an early trainee chiropractor-patient working alliance involves a process of building patients’ confidence in the trainee chiropractors’ expertise, identifying the correct goals of care, and recognising the value of the proposed treatment plan. Factors shaping this process include the context of the care journey, patients’ perceptions of trainee chiropractors’ qualities, their bodily sensations, their expectations, their past experiences, and their satisfaction with trainee chiropractors’ explanations.

**Supplementary Information:**

The online version contains supplementary material available at 10.1186/s12998-023-00527-8.

## Introduction

The clinician-patient relationship has been consistently reported to predict treatment success, regardless of the type of therapy [1, 2]. For example, a systematic review and meta-analysis of thirteen randomised controlled trials suggested that the relationship between a clinician and a patient has a small, but statistically significant effect on a range of healthcare outcomes, such as health-related quality of life, weight loss, blood pressure, pain relief, and smoking quit rate [2]. This clinician-patient relationship has been operationalised in the literature by a construct called Working Alliance (WA). Bordin developed the most comprehensive WA theory in a mental health context, but his conceptualisation is considered applicable to diverse therapies and settings, including physical health [3–5]. Bordin’s conceptualisation is designed to account for how the theory underlying a given therapy translates into a clinical change process during which the patient and the clinician work together collaboratively to reach the desired treatment outcome [3, 4]. According to Bordin, WA has three key features: (1) agreement on the goals of treatment; (2) agreement on the tasks required to achieve the goals; and (3) establishment of a bond [3, 4]. The bond does not simply reflect the overall level of rapport or respect, it also embraces the mutual trust and patients’ confidence in the treatment process [3–5].

Findings demonstrating the association between WA and subsequent psychotherapy outcomes are robust [6]. The impact of the strength of WA on outcomes is also well-documented in physical health settings [1] where WA is often measured early in treatment. For instance, a recent study found that patients’ rating of WA was a positive predictor of improvement in region specific outcome measures in patients receiving physical therapy for acute and chronic musculoskeletal pain conditions [7]. Holmes and colleagues measured WA before the third treatment session; first to ensure enough time for WA to develop and second to minimise the impact of other factors [7]. Similarly, Bishop and colleagues conducted a prospective cohort study measuring WA during the second week of treatment [8]. They found that stronger WA on all three features (bond, tasks, and goals) was a statistically significant predictor of reduced back-related disability over time in patients attending physiotherapy, osteopathy, and acupuncture clinics [8]. The impact of WA on outcomes did not differ across the three different therapies in the private and public sectors [8].

A systematic literature review concluded that patient-rated WA during treatment predicted pain reduction and improvement in physical functioning for patients with chronic musculoskeletal pain based on four cohort studies and one randomised control trial [9]. The cohort studies measured WA during the treatment intervention (e.g., during spinal manipulative therapy) [9]. The randomised control trial tested the effects on pain intensity and muscle pain sensitivity of WA as an active intervention added to interferential therapy [10]. In the control group therapists were instructed to avoid conversation during the inferential therapy treatment; in the intervention group therapists were instructed to improve WA during inferential treatment by maintaining eye contact, physical touch, offering words of encouragement, using phrases that show empathy, and asking questions to demonstrate active listening [10]. The results from the trial suggested that WA impacted pain intensity, but not muscle pain sensitivity [10]. Returning to the findings from the systematic literature review, the influence of WA on treatment outcomes was small but significant: the cohort studies indicated that WA contributed to pain reduction and physical functioning measured by means of questionnaires [9].

While most systematic literature reviews in this area assess the impact of WA on outcomes, Kinney and colleagues included studies that explored factors influencing the strength of WA [11]. Three qualitative studies used semi-structured interviews to examine factors that have positive impact on the strength of WA and one quantitative observational study explored factors that have a negative impact [11]. Examples of factors that could have positive impact were (1) a therapist working with the whole person, (2) a therapist seen as more than just a professional, (3) an individualised, flexible treatment plan, (4) the ability to work through challenges in the patient-clinician relationship, (5) rapport, (6) a trusting relationship, (7) the identification of patient values, (8) the identification of patient barriers, (9) open communication, and (10) a therapist perceived as having the patient’s best interest in mind [11]. In contrast, factors that could have a negative impact on WA according to the patients’ perspective were hostility and anger expression, and high anger expression combined with high depression according to the clinicians’ perspective [11].

Given the effect of WA on health and other outcomes, researchers should aim to understand how it develops. Like most relationships, establishing WA is almost certainly a dynamic process [4, 12]. In physical health settings, the literature on the development of WA is scarce [13, 14]. One study used a cross-case analysis to compare experiences of WA between occupational therapists and clients across four therapeutic dyads [15]. The qualitative data suggested that WA was shaped by the fostering of an interpersonal connection, a shared sense of success, and the attainment of clearly defined, patient-centred goals. These findings demonstrated the applicability of Bordin’s conceptualisation of WA in the study context [15]. However, the study characteristics - a small sample of four dyads with two community-based occupational therapies and two patients – may limit the transferability of findings to other contexts [15].

A mixed method study investigated (1) how physical therapist behaviours and interactions during the initial physical therapy assessment relate to the patient’s perception of WA and (2) the relationship between WA, pain intensity, and function [16]. Mayers and colleagues used (1) qualitative analysis to develop a checklist of WA themes and behavioural practices and (2) Spearman’s Rho (ρ) to quantify if there was an association between increased WA and improved clinical outcomes [16]. Patient-rated WA was stronger when the physical therapists used information gathering, paused to listen, used humour and transitions, and used clarifying questions [16]. Patient-rated WA was weaker when the following behavioural practices were present: lack of touch, the absence of pain neuroscience education, and not restating what the patient had said during the interview [16]. The study findings demonstrated a statistically significant negative correlation between patient-rated WA and pain intensity immediately after the initial evaluation, highlighting the relationship between early WA and clinical outcomes [16].

Most patients who seek chiropractic care do so for predominantly musculoskeletal conditions [17]. Chiropractors use a range of techniques such as hands-on manipulation of the spine, ice, heat, ultrasound, exercise, acupuncture, and advice about posture and lifestyle [17]. The qualitative component of a recent mixed methods systematic review found that both patients and chiropractors consider the features of WA to be crucial factors during care [18]. Jamison was the first to study chiropractor-patient WA: she explored the interplay between the established WA and patients’ expectations [19]. Findings from her mixed method study suggested that WA included a sense of congruence, an interpersonal bond, positive expectations, identification of shared goals, and a sense of collaboration [19]. However, that study is now over 25 years old. Moreover, the question of how WA develops during the initial stages of chiropractic care remains unclear despite most studies measuring WA in this period.

A recent modified two-round online Delphi-consensus survey was conducted with musculoskeletal practitioners to measure the extent of panel agreement on the perceived acceptability and influence of five main types of contextual factors during clinical management of patients with chronic low back pain [20]. The contextual factors of interest were (1) the patient-practitioner relationship; (2) patient’s characteristics/beliefs; (3) practitioner’s characteristics/beliefs; (4) the treatment characteristics; and (5) the treatment environment/setting [20]. The panel consisted of 23 chiropractors, 10 physiotherapists, one individual who was a qualified chiropractor and physiotherapist, and one clinical functional neurologist [20]. The findings suggest that the patient-practitioner relationship was rated as the most important contextual factor [20]. However, less than 70% of the panel reported being confident about their non-verbal communication skills and less than 70% of the panellists were confident about developing WA, expressing genuine empathy, engaging in collaborative decision-making, or requesting the patient’s opinion [20]. Conclusions from the study suggest that musculoskeletal practitioners may require further training to increase their confidence in applying essential psychosocial skills to address the complex needs of patients with chronic low back pain [20].

Even though recent research suggests WA is (1) a key predictor of outcomes in patients with low back pain [8] and (2) is identified by a consensus of chiropractors as a central factor in the delivery of chiropractic care [20], such psychosocial content is not a strong focus in either the historical identity of the profession or within existing chiropractic curricula [21]. This qualitative study explored patients’ perceptions of the initial development of WA during the early stages of chiropractic care in the context of a teaching clinic. The study aimed to understand how early WA develops between patients and trainee chiropractors to inform evidence-based practice and facilitate training development on the patient-practitioner relationship. Anecdotally, trainee chiropractors typically spend more time with patients than qualified chiropractors, especially during the initial assessment. This provides an opportunity for the establishment of WA early in the care journey, thus allowing the researchers to explore early WA development from patient’s perspective. In addition, because a diverse and large number of trainees see patients at the teaching clinic at any one time, this facilitated a systematic exploration of WA across multiple trainee chiropractors; such a breadth of sampling would have been difficult to achieve if we had worked with qualified chiropractors (who in the United Kingdom (UK) mainly work in single-handed or small practices) [22]. Finally, the subsidisation of fees in a teaching clinic meant we were able to examine WA in the context of a more diverse patient population.

## Methodology

### Design

This qualitative study explored patients’ perspectives on the formation of a relationship between themselves and trainee chiropractors early in the care journey. The study was approved by the Faculty Ethics Committee at the University of Southampton (Number: 74,271). The study was conducted in the teaching clinics at AECC University College (AECCUC), which is a specialist health care professions training university in UK [23]. In this setting, health care trainees work under the supervision of qualified academic clinicians. The qualitative methodology used in this study gave patients voice by allowing the researcher to explore their interpretation of the development of WA [24]. The use of semi-structured interviews addressed patients’ perspectives on the complexity underlying the formation of WA, which in turn may inform an in-depth understanding of how early WA can influence outcomes [12].

The researchers considered and discussed their stance on ontology (the nature of reality), epistemology (our knowledge of reality) and the implications of such assumptions on the study and its findings [25]. The philosophical stance chosen to underpin the study was critical realism [26]. Ontologically, critical realism assumes that the nature of reality is not reducible to our knowledge of it; in other words, an external reality exists, and it is independent of our perception of it [26]. According to critical realism, there is a difference between experiences (how patients interpret their relationship with the chiropractor), events (the development of a relationship during care), and causal tendencies (factors that impact the formation of a relationship) [26, 27]. Generally, patients’ perceptions of WA are highly subjective experiences comprised of emotional and cognitive dimensions that include appraisals of clinicians’ intentions and the perceived value of treatment [12]. From a critical realist standpoint, patients may not necessarily be fully aware of the causal tendencies or conditions underlying the formation of WA with chiropractors. Instead, qualitative interviews provide insight into patients’ interpretations of their experiences [26, 27]. To understand how WA develops, the researcher further considered the context in which the study was conducted and drew on their knowledge of psychological theories and evidence to account for the findings.

### Participants

Participants were recruited from a patient pool enrolled in an online longitudinal questionnaire at AECCUC study via availability sampling during September 2022 and April 2023. For the purpose of this study, the AECCUC was sometimes also referred to as “the college”, because participants used this term to refer to the AECCUC. The inclusion criteria for patients were as follows: (1) adult (at least 18 years) seeking chiropractic care at the AECCUC teaching clinic; (2) receiving treatment from a chiropractic student trainee who will be available for the whole duration of treatment; and (3) has not visited AECCUC in the last one year. The purpose of Criterion 3 was to ensure that a potential participant would be working with a trainee for the first time so that the formation of WA could be studied from the beginning. The exclusion criteria were as follows: (1) patients lacking full mental capacity, and (2) inability to complete questionnaires and interview in English. Pain duration and severity, co-morbidities and co-treatments were recorded to describe the sample.

The authors discussed in advance the use of saturation in the context of Reflexive Thematic Analysis (RTA), which is the method of data analysis used in this study [28]. Braun and Clarke, the scholars who developed RTA, contend that concepts such as data saturation, thematic saturation, code saturation, and even meaning saturation are not in line with the values and assumptions of RTA [29]. We followed the model developed by Malterud and colleagues to guide the sample size of the study [30]. According to the model, the more information relevant to the research aim a sample holds, the smaller number of participants is required [30]. The sample size depends on the following elements of information: (1) the aim of the study, (2) sample specificity, (3) use of established theory, (4) quality dialogue, and (5) analysis strategy [30]. On one hand, the study aim was based on established theory, which reduced the required sample size. On the other hand, there were four interviews which lasted around 10 min, which did not allow for a high-quality dialogue between interviewer and participants, thus increasing the required sample size. Considering further the aim of the study, the sample specificity, and the analysis strategy, we agreed that a sample of 25 participants provided sufficient information power to explore early WA development between patients and trainee chiropractors.

A total of 53 patients from the pool of 71 participants in the longitudinal questionnaire study gave an online consent to be contacted regarding the qualitative study. Out of the 53 patients, a total of 25 participants (12 females and 13 males) took part in the qualitative study and completed an interview. The majority were English, Welsh, Scottish, Northern Irish, or British (*N = 22*); the remaining three participants were White Asian, Spanish, and French. Twelve patients had never received chiropractic care before, five had received chiropractic care at AECCUC more than a year ago, and eight had received chiropractic care in a different clinic. The main reasons for visiting the AECCUC were back pain (*N = 11*), shoulder pain (*N = 6*), neck pain (*N = 3*), knee pain (*N = 2*), general wellness (*N = 2*) and unspecified (*N = 1*). The median age of the group was 55. Twenty one participants experienced pain with a median score of 6 on a 10-point numerical rating scale (Table 1). Five participants had chronic conditions: three of them said that their condition limited their activities.

**Table 1:** Age and Pain Severity

	N	Minimum	Maximum	Median
Severity of pain	21	2.0	9.6	6
Age	25	24	78	55

### Semi-Structured Interviews

On enrolling in a longitudinal questionnaire study, patients were asked if they would be interested in taking part in this qualitative study. Patients who expressed interest in the qualitative study were invited to participate in one semi-structured interview early in participants’ care. Interviews were conducted by the first author (DI) who is a female PhD researcher in Psychology at the University of Southampton with MSc in Social Research Methods. The interviewer had no established relationship with the participants prior to study commencement. The interviews lasted between 10 and 40 min with an average duration of 23 min. They were conducted synchronously without cameras on a one-to-one basis via Microsoft Teams software (version 1.5.00.28361) [31] and were audio-recorded with participants’ informed consent. They consisted of open-ended questions, thereby allowing participants to share their experiences: starting with broad questions about patients’ experiences, followed by specific questions about their expectations and their perception of collaboration, communication, and a potential bond between them and the trainee chiropractor. The topic guide (Supplementary File 1) was developed by DI under the supervision of the co-authors and was informed by Bordin’s conceptualisation of WA and the findings of a published systematic literature review exploring the nature of WA in the chiropractic profession [3, 4, 18]. The interview guide was pilot tested with one individual, who was not a participant in the study.

### Data Analysis

Interviews were transcribed verbatim using the transcripts provided by Microsoft Teams as a starting point and then and checked for accuracy. The interview transcripts were analysed by using RTA [32] in NVivo (Version 12) [33]. This qualitative method involves a reflexive engagement with the data and follows a six-phase recursive process. It emphasises both accurate reflection of the qualitative data and the active role of the researcher in the knowledge production process [28, 34]. RTA was used because the method acknowledges the role of the researcher’s subjectivity as a resource, thereby enabling interpretation of patients’ experiences in relation to available literature and the study context [28, 34]. The six phases of RTA outlined by Braun and Clarke follow a sequential order but involve moving back and forth between phases [28].

The first two phases included familiarisation with the dataset and coding [28]. The researcher (DI) read and reread the transcripts, saving initial analytic insights as memos and annotations [28]. During coding, semantic, and latent codes were generated using inductive and deductive orientations, respectively [28]. In the former, the analytic process started from the data itself [27]. In the latter, the researcher (DI) approached the data with preconceived ideas and concepts that reflected the study context and relevant theory, including social cognitive theory and Bordin’s theory of WA [3, 27, 28, 35]. The next two phases included the generation, development, and revision of themes [28]. After DI generated the initial themes, FLB, DN, and JF participated in the last three phases of the reflexive thematic analysis, which included developing and reviewing, defining, and naming the themes, and finally, the production of the report. FLB is a Professor of Health Psychology with extensive experience in qualitative research. DN is a Professor of Integrated Musculoskeletal Care with extensive experience in chiropractic profession. JF is a highly experienced Chiropractic Clinician with a PhD from University of Portsmouth. A critical realist approach to RTA translated the philosophical stance into methodological practice by using three types of themes: experiential, inferential, and dispositional themes [27]. The experiential themes were data-driven and referred to participants’ subjective viewpoints, describing their experiences, feelings, hopes and concerns [27]. Inferential themes were derived from the experiential themes and included the researchers’ inferences and conceptual redescriptions of participants’ experiences [27]. Finally, the dispositional themes were derived from the inferential themes and reflected theories about potential causal tendencies underlying the formation of early WA. During the final phase, the production of the report, the researchers continued referring to the research question, the lists of codes, and the theme definitions to ensure that the finalised dispositional four themes addressed the research aims comprehensively [27]. The participants did not provide feedback on the finalised themes.

## Results

The first step of early WA development involves a process of building participants’ confidence in their choice to put their health in the hands of the trainee chiropractor. The following quote from Participant 8 can be mapped to the three features of WA and suggests that at the start of care, the participant wanted to feel confident in the expertise of the trainee chiropractor before developing a bond with them. To reach agreement on the goals of care and the proposed plan, the participant also wanted to feel confident that their treatment was appropriate and designed to deal with a correctly identified problem.

*‘I need to know that they know what they’re doing. They know what they’re talking about. And I’ve got to be in a situation where I believe that what they’ve told me is the answer to the problem, and not that they’re just having a good guess. So, I need to be convinced that what they’ve said needs to be done is actually what needs to be done.’ (Participant 8).*

Generally, at the start of the chiropractor-patient relationship, participants wanted trainee chiropractors to make them feel confident in the upcoming care journey. The four themes reveal the factors that impacted the extent to which participants felt confident and thus shaped early WA. Table 2 provides a summary of the themes and their key attributes.

**Table 2:** Themes – Summary and Key Attributes

Theme Name	Key Attributes
*The Context of the Clinic: Setting up the Conditions for the Process of Building Patients’ Confidence in the Upcoming Care Journey*	1) The role of clinic’s reputation and others’ recommendations in participants’ openness to initiating working alliance. 2) The role of the thorough assessment. 3) The role of tutors’ supervision.
*Key Chiropractors’ Qualities: Confidence and Perceived Expertise in the Context of Early Working Alliance*	1) Trainee chiropractors’ self-confidence. 2) Trainee chiropractors’ perceived expertise. 3) The impact of patients’ sense of touch on their perception of trainee chiropractors’ key qualities.
*The Role of Explanations in the Development of Working Alliance*	1) The role of trainee chiropractors’ explanations in the process of establishing agreement on treatment goals and plan. 2) The role of trainee chiropractors’ explanations in the process of establishing a bond, consisting of trust, confidence and mutual understanding. 3) The potential role of trainee chiropractors’ explanations in patients’ adherence to the treatment plan. 4) Active listening and clear explanations as tools to amend differences in opinions.
*Working Alliance in the Context of Pain*	1) Participants describe a sense of vulnerability during chiropractic care. 2) The importance of active listening and the established bond in the context of patients’ experience of chronic pain. 3) Patients’ experience of pain in the context of treatment goals. 4) The importance of trainee chiropractor-patient agreement in the context of potentially painful situations.

### Theme: The context of the clinic - setting up the conditions to build patients’ confidence in the upcoming care journey

*‘The attitude of AECCUC is that they don’t want to see you forever. So, it’s not just about managing the pain, or fixing the problem. It’s about the long-term journey, how do you then maintain the issue, and not just fix it. How do we fix it and then maintain it, so that’s important.’ (Participant 10).*

A shared belief across most participants was that the AECCUC is a well-respected institution in the locality and region that would provide them with high quality care. This theme captures four main ways in which the context of the clinic shaped early WA development.

First, participants shared that their expectations were raised because of the people they had spoken to or the success of their previous visits. An existing positive opinion can make patients more inclined to trust the trainee chiropractor and thus more open to initiating WA. In contrast, Participant 7, who had a previous negative experience at AECCUC, shared that the initial trust towards the trainee chiropractor was “*about 60%*”, but they expected this to grow with time.

Second, most participants noted having a thorough assessment, which is standard practice during the first visit at the clinic and typically lasts two hours. For example, the thoroughness of the assessment left Participant 10 with the impression that the attitude of AECCUC was “*a lot more personal*” compared to other qualified chiropractors, who can be “*one in, one out*”. This two-hour assessment gave participants a sense of reassurance, which had a positive impact on the process of building their confidence. Arguably, the time spent examining potential causes of their symptoms convinced participants that the proposed treatment would be appropriate. Furthermore, some participants explained that while going through the assessment, the trainee chiropractors demonstrated their background knowledge, which increased participants’ confidence in the trainee chiropractor. For example, Participant 19 explained that the initial assessment was “*the platform from which the confidence initially was very well established but then built on and maintained*”.

Third, most participants acknowledged that the trainee chiropractors at AECCUC were still in the process of learning. As a result, participants adjusted their expectations, and in some cases, they even empathised with their trainee chiropractor. This was mainly evident in participants’ acknowledgement that the trainee chiropractors may lack self-confidence and display some nervousness, but this did not impact their impression of the trainee chiropractors’ capabilities. Interestingly, Participant 7 reflected on a past experience at AECCUC, emphasising that they should have escalated a problem to the tutor. They further explained that they did not do so because they did not want to *“get the trainee chiropractor in trouble.”*

Finally, most participants described expecting that the trainee chiropractor should discuss everything with a tutor. Participants wanted to know that the trainee chiropractor and the tutor agreed on the treatment plan. It could be argued that participants’ knowledge of the role of tutors’ supervision was crucial in the process of building their confidence in the upcoming care journey. In summary, the context of the clinic helped to shape early WA development. The next theme explores which of the trainee chiropractors’ qualities the participants considered important for early WA.

### Theme: Key chiropractor qualities - confidence and perceived expertise in the context of early working alliance

Early WA included a process of building patients’ confidence in the expertise of the trainee chiropractor. Interestingly, participants deemed self-confidence to be one of the most important qualities of a good chiropractor. Overall, the interviews indicated that when trainee chiropractors show self-confidence, this self-confidence could make participants feel more confident in their expertise. Participant 9 explained that healthcare professionals should have “*some kind of a pleasant authority, where they command the respect and confidence of the patient*”. According to the participant, self-confidence was evident in the style of communication: ‘…It’s absolutely about communication…the combination of service, assertiveness, arrogance in the nicest possible way. In other words, “I know what I’m doing” kind of attitude, you know, “I’m telling you, *patient’s name*, this is what you need to do.” That kind of thing. You know, we all need that at some times…’.

While some trainee chiropractors were more self-confident than others, participants took into account that the trainee chiropractors were still in the process of learning. Overconfidence was viewed unfavourably. The following quote illustrates the impact of the clinical context on patients’ expectations about chiropractors’ self-confidence:

*‘I just felt completely trusting in and everything that they did. It’s something I don’t think you could quantify it. If he was nervous, it didn’t really show and they came across as confident but also not overconfident, I think that that would be in that situation, just as bad, if someone became brash and instantly said to me “oh, we know exactly what the problem is, come back and we’ll fix it.” That actually wouldn’t have made me confident. But the feedback and the understanding of seeing all those copious notes taken and then the fact that summary is going to be given and a plan…. that’s what’s given me the confidence that it will be sorted and it’s well worth I made the right decision to go there.’ (Participant 6).*

Furthermore, when trainee chiropractors display self-confidence, they can help patients feel less tense, potentially facilitating manual intervention. For example, Participant 7 reflected on their expectations: “*I suppose they (trainee chiropractors) need to feel relaxed enough with the patient, sometimes if you’re very tense and you talk to somebody you, you show that you tense and make them tense as well.*” Participant 7 explained in more detail what they felt was important to get along with their trainee chiropractor: “*I would say confidence. Ability to put me at ease… Someone who looks like they know what they’re doing. You know, you can know what you’re doing, but knowing it and having the confidence at the same time”* (Participant 7). Similarly, Participant 5 clarified:

*“if you haven’t got good working relationship and people are trying to manipulate you, you’re probably already a little bit but more rigid to begin with*”.

Interestingly, participants assessed trainee chiropractors’ self-confidence by considering their own sense of touch. Participant 24 compared the touch of the trainee with the touch of the tutor during examination. While the former was “*a little bit more tentative*”, the latter “*just went straight in – very confident, very firm*”. Similarly, Participant 22 shared that they wanted their trainee chiropractor to be less gentle: “*If I don’t feel the pain, I feel that the treatment is not enough*”. We argue that the more self-confident the trainee chiropractor was, the more convinced the patient was of their expertise. The following quote suggested that a participant may feel confident in the chiropractors’ expertise simply based on the degree of self-confidence perceived, independently of their actual knowledge and skills:

*‘I never had the same confidence that he was going to work. His predecessor managed to exude confidence. Certainly, that the chap I’m seeing now, he seems to be the same. It’s a bit like the captain of the aeroplane you’re flying on, the doctor at the surgery you have, we are brought up to have complete faith in these people. And only when the plane crashes, we realised we were wrong, but you know.’ (Participant 9).*

In addition to noticing trainee chiropractors’ self-confidence, participants also judged their expertise in the early stages of developing WA. When asked about their first impression of the trainee chiropractor, most participants referred to their perception of the trainee chiropractor’s knowledge and skills. The phrase “*seemed to know what they were doing*” in the context of trainee chiropractors’ perceived expertise was quite common across the interviews. Participant 1 said that they respected the trainee chiropractor because of their perceived expertise: “*I’ve got respect for him because he knows what he’s doing*”. Similarly, Participant 24 wanted to be feel confident that, indeed, the trainee chiropractor would know what they were supposed to do: “*it’s an expectation that they are the professional so you come to them for help … I’d like to feel that they know what they’re doing*”. Similar to perceived self-confidence, a trainee chiropractor’s perceived expertise may impact the extent to which patients physically relax during manual intervention.

Participants’ interpretation was that the tenderness during palpation suggested that the trainee chiropractor knows what they are doing. Participant 7 described how the trainee chiropractor was trying to reproduce the pain during examination based on what the participant had shared. In this case, the elicited tenderness demonstrated to them that the trainee chiropractor understood them correctly. Likewise, Participant 8 shared how their trainee chiropractor explained the notion of referred pain: “*She was able to poke one side and make it hurt on the other side, which wasn’t something that I encountered. And it made me realise that, obviously, she knew what she was talking about”.* This theme focused on one aspect of early WA development – building patients’ confidence in the trainee chiropractors’ expertise. Another aspect of early WA is discussed in more detail in the next theme.

### Theme: The role of explanations in the development of working alliance

Generally, explanations were the key factor impacting the process of early WA development and they appeared to do this in four main ways.

First, explanations fostered trainee chiropractor-patient agreement on the goals of care and collaboration on the treatment plan through negotiation. For example, when Participant 6 was asked about factors that could help them feel involved in the decision-making process during care, they noted the role of explanations:

*‘I think if they explain clearly what it is we need to do and perhaps what is a likely cause of the problem, I think they could then explain to me the causes and then what can be done not only from their point of view in terms of manipulation or whatever they do, but they can then explain the need for me to do certain things. And maybe I’ve been doing something for a long time which builds up and causes the problem. Then it would be the case of them explaining to me clearly what’s the problem and I’m sure we can then openly talk. And then I can realise: “Oh yes, I’ve been doing that” or “I’ve always been sitting in a certain way”.’ (Participant 6)*.

Second, explanations can enable patients to relate to and bond with the trainee chiropractor by helping them understand the trainee chiropractor’s thinking behind the proposed treatment plan. This in turn can build patients’ confidence in the upcoming care journey. For example, Participant 8 reflected on the need for satisfactory explanations: ‘…*I would need to feel comfortable having spoken at length about it, having been tended to by the student. Then the student having gone away and thought about it and discussed it with a tutor has come back and said: “Well the answer is that and that and what we need to do is this this and this”. I’m very comfortable. But I would be a bit concerned if I thought: “Well, that did not make any sense to me”. In this case I’m very convinced that she’s got the answer….’*. Similarly, referring to the development of a trainee chiropractor-patient bond, Participant 7 shared that if a trainee chiropractor did not explain to them what they wanted and did not help them find the way to achieve what they needed to achieve, they would lose trust. Participant 5 noted that “*if you don’t trust your chiropractor, you’re not really going to be invested in what they’re trying to do and what they’re thinking. I think on a more formal aspect, if you have a good working relationship you’re more inclined to follow the instructions they’ve given you, if they’re giving you rehabilitation at home.”.* On one hand, satisfactory explanations could impact the development of mutual trust, and on the other hand, the established trust increased participants’ receptiveness to the explanations provided by the trainee chiropractors.

Third, explanations can enable appropriate adherence to the treatment plan and potentially facilitate recommended lifestyle changes. For instance, Participant 10 shared that they wanted a chiropractor to demonstrate how to do an exercise, and then observe them doing it to ensure that it was done properly. This “*feedback loop*”, as Participant 10 described, can help patients feel reassured that they know how to follow the treatment plan correctly. Chiropractors’ explanations could help patients learn new insights about their body, which in some cases led to the immediate implementation of a lifestyle change. In the context of lifestyle changes, Participant 11 explained “*I’m clear there’s a lot of patience from the practitioner because I am quite interested in this type of thing, chiropractic care and how it works and just to aid myself as well, because, of course, my practitioner is treating, but I got the lifestyle changes to adjust, perhaps, if required.”* Explanations can help patients to understand how to implement potential changes in their life and follow the recommended treatment plan.

Fourth, explanations can be used as a tool to amend differences in opinions. For instance, Participant 4 and their trainee chiropractor had a different interpretation of the participant’s blood pressure score. For the trainee chiropractor, the score was healthy for the patient’s age, but for the participant, the score was high compared to their normal range. Participant 4 noted that “*the conversation needs to be flowing and you need to feel that you are the expert of how you feel about something and your experience of it*”. In cases where the trainee chiropractor and the patient have a mismatch in their interpretations of a situation, explanations can potentially help both sides to clarify any misunderstanding. The importance of explanations did not only concern the explanations provided by the trainee chiropractors. Participants wanted to feel that their own explanations were being listened to, considered, and validated, particularly in potentially vulnerable or painful situations. The next theme explores the role of patients’ pain in early WA development.

### Theme: Working alliance in the context of pain

As discussed, early WA development included a process of building patients’ confidence in: (1) the expertise and trustworthiness of the chiropractor, (2) the correctly identified problem causing patients’ symptoms and (3) the appropriateness of the proposed treatment plan. The importance of the three features of WA was especially evident in the context of pain.

The interviews revealed that participants experienced a sense of vulnerability during chiropractic care, which increased the need for established WA, especially in potentially painful situations. For example, Participant 5 noted the role of trust when a patient is in a vulnerable position: “*I think you (a trainee chiropractor) have to have good communication skills, especially with the more vulnerable people… they are putting their health in your hands really, especially when they’re using manipulation, chiropractors have the ability to cause harm so there has to be that element of trust. Putting people at ease is probably a big part of that”.* Participant 12 reflected further on the role of WA in cases when a patient feels stressed: *“I think when you’re under a bit of stress in those sort of situations, you just need to feel comfortable, which I do…I trust what she’s trying to achieve and it’s all being done with the right goals at the end*”. Participant 16 also explained: *”I think when somebody sort of working on your body anyway, I think it’s important to feel comfortable. If you don’t get along with somebody, I think it can be quite uncomfortable having that closeness with somebody.”* This theme explores the interplay between WA and participants’ experience of pain and vulnerability.

First, Participant 22 shared their thoughts on their chronic pain, illustrating the importance of the established bond, capturing patients’ confidence in trainee chiropractors’ expertise and trustworthiness:

*‘With my chiropractor, I was so relaxed because I knew I could trust her with whatever she was going to do with my body, because she gave me that trust right from the assessment. Now, if someone was rough on the assessment and wanted to move on to one question to another to another… you don’t want to listen to me and my pain, my chronic pain, because they are real pains…. I’m not going for a treatment just for someone to get some money out of me.’ (Participant 22)*.

Participant 22 wanted to feel that the trainees truly cared about them and their pain. Generally, participants wanted to feel validated in the context of their experience of pain. This is where the role of active listening was most crucial. For example, Participant 7 commented further on what would make them lose trust in the trainee chiropractor: “*If they’re not validating what I’m saying. If they’re not listening, if I’m saying that I’m in pain or it doesn’t work, or I can’t do it, or I don’t feel the stretch or it doesn’t do anything for my muscle*s”. Some participants highlighted that they needed to trust the trainee chiropractor to let them relieve their pain. They realised that the established trust could have both psychological and physiological impacts. Participants acknowledged that the more they trusted the trainee chiropractor, the more comfortable they felt during manual intervention (psychological impact). Additionally, the more relaxed their body was during manual intervention, the more they could get out of it, because their body was less tense (physiological impact).

Second, pain played a role in the process of identifying the problem, which the proposed treatment plan was supposed to address. The participants revealed that sharing about and describing their pain can be challenging. For instance, Participant 20 shared that it was difficult to accurately explain the sensation in their body; thus, they felt that the trainee chiropractor did not understand them completely. This can impact the mutual agreement treatment goals. In contrast, Participant 7 explained how they could not make sense of the pain in their body until the trainee chiropractor validated their experience by providing a physiological explanation.

Third, participants valued the mutual agreement on the proposed treatment plan. They wanted to feel confident in the trainee chiropractors’ readiness to be receptive of their needs, demonstrating empathy and patience. For example, Participant 7 explained: *“If I’m saying I can’t do that because it hurts too much, or I could do it, but it hurts too much the next day, then I want them to take that into consideration.”.* The discomfort of patients’ pain may amplify the need for establishing WA, especially in regard to reaching agreement on how to alleviate their symptoms. Participant 11 described how their trainee chiropractor engaged in an ongoing negotiation with them in a potentially painful situation:

*“She’s always asking me to let her know about my pain between one and ten. So when she’s prodding, for example, and I’m communicating. So we have this open dialogue, so if I say two, two is fine, it’s totally manageable. But if I was someone had more pain, then that could be very important because you tense as well, don’t you, when you think something is going to hurt. And of course, to get the best out of the whole treatment, you’ve got to be relaxed, really. And that could be a difficult thing if you don’t have confidence in your practitioner”.*

Overall, participants’ experience of pain and the related sense of vulnerability (1) amplified the need for established confidence in the trainee chiropractors’ trustworthiness and expertise, (2) shaped the process of establishing agreement on treatment goals, and (3) emphasised the need for trainee chiropractors’ receptiveness to participants’ feedback on the treatment plan.

## Discussion

### Summary of Findings

The results from the thematic analysis illustrated the development of chiropractor-patient WA from the perspective of 25 participants in the initial stages of their care with trainee chiropractors. The findings emphasised that, at the start of care, WA involved a process of building participants’ confidence in their choice to seek trainee chiropractor’s help to alleviate their symptoms. When assessing their own level of confidence in the upcoming care journey, participants considered the recommendations from trusted others, the thorough assessment in the clinic, and the reassurance that tutors and trainees agree on the proposed treatment. Additionally, they referred to their bodily sensations, their perception of the trainee chiropractors’ self-confidence and expertise, and the provided explanations. Reflecting on the described sense of vulnerability related to chiropractic care, participants emphasised that trainee chiropractors should validate their experience of pain by listening to and considering their needs, explanations, concerns, and preferences. The four themes described (1) the impact of the context of the clinic on participants’ expectations, (2) the trainee chiropractors’ qualities that participants considered important, (3) the role of explanations, and (4) WA importance in the context of pain. The next sections will discuss how the themes relate to the wider literature.

The participants in this study were recruited from a patient pool at the teaching clinic set in a specialist health care professions training university in the United Kingdom. The context of the clinic impacted early WA development in the following ways. First, the university was a well-respected institution in the locality and region. This contributed to participants’ positive expectations which were partly based on the reputation of the clinic. Second, while the interviews suggested that trainee chiropractors’ self-confidence can play a role in early WA development, participants acknowledged that the trainee chiropractors were still in the process of learning. Thus, participants adjusted their expectations accordingly, accepting that the trainee chiropractors may show some nervousness. However, our findings further demonstrated the importance of recommendations from trusted others, patients’ perception of trainee chiropractors’ self-confidence and expertise, and the role of explanations. These findings are in line with the research outlined in the next paragraphs, suggesting that the results of the thematic analysis provide a plausible explanation of early WA development between the trainee chiropractors and their patients.

The interviews illustrated how patients’ expectations were shaped by their own previous experiences and recommendations from trusted others. Similarly, in an ethnographic case study exploring the development of trust between a chiropractor and his patients, Bolton illustrated the role of expectations in the chiropractic-patient relationship [36]. According to the author, there are prior conditions that influence how patients define this relationship [36]. On one hand, the relationship is similar to other everyday encounters because it embraces interpersonal expectations, private agendas, and social conventions [36]. On the other hand, the relationship has a predetermined purpose - improving patients’ circumstances [36]. It is further characterised by the mutually recognised discrepancy in the knowledge and power of the parties involved [36]. Bolton suggested that when a patient first books an appointment, their hope for improvement is not based on the personal or professional characteristics of the chiropractor. Instead, their hope is based on their beliefs about the authenticity of the expertise which the chiropractor is thought to embody [36]. In other words, there are prior expectations of the expertise of a chiropractor which define the chiropractor-patient relationship before the two parties have even met. Likewise, in our study, most patients had already formed positive expectations prior to the chiropractor-patient meeting. After the two parties met, early WA was influenced by (1) trainee chiropractors’ ability to display self-confidence and (2) trainee chiropractors’ ability to build patients’ recognition of their professional expertise.

The impact of others’ recommendations and patients’ own perception of practitioners’ expertise was also evident in a mixed method study in acupuncture clinics [37]. First, the authors found that the participants prioritised recommendations when deciding to consult a particular acupuncturist [37]. Second, the findings revealed that potential patients considered qualities such as trustworthiness and expertise to be important [37]. In our study, trainee chiropractors’ self-confidence could impact how a patient perceived their expertise. This is important, because expertise has been previously reported as a quality that patients prioritise. For example, the findings of an integrative review suggested that a ‘good’ musculoskeletal physiotherapist should be responsive, ethical, communicative, caring, competent, and collaborative [38]. In the context of this study, the extent to which patients felt confident in the trainee chiropractor’s expertise and trustworthiness appeared to shape the process of establishing agreement on the goals of care and the proposed treatment plan. Similarly, the findings from a qualitative study exploring patients’ expectations before and experiences after physical therapy for low back pain described how participants anticipated the therapist to be the body-oriented expert who can give an accurate diagnosis [39].

There were rules of treatment that followed from the education that the trainee chiropractors received in their clinical placement at university. In line with Bordin’s conceptualisation of WA, the education underlying chiropractic care sets out certain expectations and demands for the chiropractor and the patient and how they work together to effect change in patients’ circumstances [3, 4]. The extent to which both parties work together following these rules can impact the extent to which change occurs [3, 4]. The role of WA is to ensure successful negotiation between the trainee chiropractors’ expectations, which are guided by their education, and those of the patients, reflecting their own beliefs and understanding of their health [3, 4]. The three key features of WA, namely, agreement on the goals of care, collaboration on the treatment plan, and the establishment of bonds, facilitate this negotiation.

Our findings suggest that a key factor in early WA development was the role of explanations. To establish chiropractor-patient agreement on the treatment plan, the trainee chiropractors in this study utilised explanations and demonstrations, ensuring that participants felt confident that their treatment was appropriate and designed to deal with a correctly identified problem. The importance of explanations was also illustrated in the results of a mixed method systematic review exploring WA in the chiropractic literature [18]. The findings described how patients tend to view chiropractic care as a change process whose end goal is improvement of their circumstances. It could be argued that the role of the chiropractor is that of a change agent [18]. Satisfactory explanations can ensure that the patients understand the need for a change. In our study, trainee chiropractors’ explanations could help patients relate to them, learn new insights about their body, understand the reasoning behind the proposed treatment plan, and realise the need for potential lifestyle changes.

Furthermore, trainee chiropractors’ demonstrations of the required exercise regime built patients’ confidence and provided a sense of reassurance that they could correctly follow the treatment plan or the recommended lifestyle changes. The findings of a longitudinal qualitative study examining WA from the perspective of patients receiving physical therapy illustrated how patients experienced the treatment as empowering [39]. This perception arose from the physiotherapists’ effective communication of their expertise through the use of explanations [39]. Previous research suggested that one possible explanation of the impact of WA on outcomes is the positive correlation between adherence and WA. For example, a longitudinal qualitative study with older adults with knee pain investigated the relationship between WA and adherence [40]. The study used semi-structured interviews with 30 participants at baseline, who had been randomised to one of three physiotherapist-led exercise intervention arms in a clinical trial [40]. The findings indicated that strong WA is a key facilitator of exercise adherence in the short and longer term regardless of the intervention arm [40]. Participants appreciated physiotherapists who took time to get to know them, understand them, empathise with them, and last but not least, explain and show them the exercises [40]. Similarly, Fuertes and colleagues examined psychological and behavioural sequalae of WA among patients with diverse health conditions including HIV+/AIDS, hypertension, diabetes, asthma, and cancer [41]. They found that stronger patient-rated WA and higher self-efficacy both predicted higher patient adherence to treatment, while stronger WA also predicted greater patient satisfaction [41].

### Study Limitations, Research Opportunities and Recommendations

This study explored the formation of WA at the start of chiropractic care from the perspective of 25 patients attending AECCUC undergoing care from trainee chiropractors. To gain a full picture of how WA is established, future research should explore chiropractors’ perspectives and the development of WA within cohorts of qualified chiropractors. Additionally, research including participants from different ethnic backgrounds could help explore the role of cultural differences in early WA development. Considering the impact of the clinical setting on patients’ expectations, the findings in the theme “The Context of the Clinic: Setting up the Conditions for the Process of Building Patients’ Confidence in the Upcoming Care Journey” are not directly transferable to non-training chiropractic settings. Instead, the findings revealed how context can impact the development of WA. While this study was focused on the early stages, future research should advance our understanding of how WA progresses with time. Our findings did not reveal any major disruptions in WA between the trainee chiropractors and the participants. However, a research opportunity that can inform clinical practice is exploring how chiropractors amend disruptions in WA. Furthermore, the study findings demonstrated that established WA was especially important in the context of potentially painful situations, namely during pain assessment and manual intervention. A more in-depth understanding of the relationship between touch, patients’ experience of pain, and WA could provide potential recommendations for clinical practice. A potential limitation of our study is that due to practical constraints, interview transcripts and finalised themes were not returned to participants to ensure accuracy.

## Conclusions

Our findings described early WA development between trainee chiropractors and their patients, which involved a process of building patients’ confidence in the trainee chiropractors’ expertise, the correctly identified goals of care, and the value of the proposed treatment plan. Factors that shaped the process of establishing early WA included the context of the care journey, patients’ perception of trainee chiropractors’ qualities, and their satisfaction with trainee chiropractors’ explanations. Patients further consider their bodily sensations, their expectations, and past experiences.

### Electronic supplementary material

Below is the link to the electronic supplementary material.

**Supplementary Material 1:** Topic Guide

**Supplementary Material 2:** Checklist

**Supplementary Material 3:** COREQ 32-Item Checklist

## Data Availability

The datasets generated and analysed during the current study are not publicly available due confidentiality reasons but the codebook is available from the corresponding author on reasonable request.

## References

[1] Hall AM, Ferreira PH, Maher CG, Latimer J, Ferreira ML (2010). The influence of the therapist-patient relationship on treatment outcome in physical rehabilitation: a systematic review. Phys Ther.

[2] Kelley JM, Kraft-Todd G, Schapira L, Kossowsky J, Riess H (2014). The influence of the patient-clinician relationship on healthcare outcomes: a systematic review and meta-analysis of randomized controlled trials. PLoS ONE.

[3] Bordin ES (1979). The generalizability of the psychoanalytic concept of the working alliance. Psychotherapy: Theory Research & Practice.

[4] Bordin ES. Theory and research on the therapeutic working alliance: New directions. 1994.

[5] Elvins R, Green J (2008). The conceptualization and measurement of therapeutic alliance: an empirical review. Clin Psychol Rev.

[6] Flückiger C, Del Re AC, Wampold BE, Horvath AO (2018). The alliance in adult psychotherapy: a meta-analytic synthesis. Psychotherapy.

[7] Holmes MB, Scott A, Camarinos J, Marinko L, George SZ (2023). Working Alliance Inventory (WAI) and its relationship to patient-reported outcomes in painful musculoskeletal conditions. Disabil Rehabil.

[8] Bishop F, Al-Abbadey M, Roberts L, MacPherson H, Stuart B, Carnes D (2021). Direct and mediated effects of treatment context on low back pain outcome: a prospective cohort study. BMJ open.

[9] Lakke SE, Meerman S (2016). Does working alliance have an influence on pain and physical functioning in patients with chronic musculoskeletal pain; a systematic review. J Compassionate Health Care.

[10] Fuentes J, Armijo-Olivo S, Funabashi M, Miciak M, Dick B, Warren S (2014). Enhanced therapeutic alliance modulates pain intensity and muscle pain sensitivity in patients with chronic low back pain: an experimental controlled study. Phys Ther.

[11] Kinney M, Seider J, Beaty AF, Coughlin K, Dyal M, Clewley D (2020). The impact of therapeutic alliance in physical therapy for chronic musculoskeletal pain: a systematic review of the literature. Physiother Theory Pract.

[12] Muran JC, Barber JP. The therapeutic alliance: an evidence-based guide to practice. Guilford Press; 2011.

[13] Babatunde F, MacDermid J, MacIntyre N (2017). Characteristics of therapeutic alliance in musculoskeletal physiotherapy and occupational therapy practice: a scoping review of the literature. BMC Health Serv Res.

[14] Horton A, Hebson G, Holman D (2021). A longitudinal study of the turning points and trajectories of therapeutic relationship development in occupational and physical therapy. BMC Health Serv Res.

[15] Morrison TL, Smith JD (2013). Working alliance development in occupational therapy: a cross-case analysis. Aust Occup Ther J.

[16] Myers C, Thompson G, Hughey L, Young JL, Rhon DI, Rentmeester C (2022). An exploration of clinical variables that enhance therapeutic alliance in patients seeking care for musculoskeletal pain: a mixed methods approach. Musculoskelet Care.

[17] Beliveau PJ, Wong JJ, Sutton DA, Simon NB, Bussières AE, Mior SA (2017). The chiropractic profession: a scoping review of utilization rates, reasons for seeking care, patient profiles, and care provided. Chiropr Man Ther.

[18] Ivanova D, Bishop FL, Newell D, Field J, Walsh M (2022). Mixed methods systematic review of the literature base exploring working alliance in the chiropractic profession. Chiropr Man Ther.

[19] Jamison J (1996). The chiropractic consultation: establishing a therapeutic alliance. Chiropr J AUSTRALIA.

[20] Sherriff B, Clark C, Killingback C, Newell D (2023). Musculoskeletal practitioners’ perceptions of contextual factors that may influence chronic low back pain outcomes: a modified Delphi study. Chiropr Man Ther.

[21] Gliedt JA, Battaglia PJ, Holmes BD (2020). The prevalence of psychosocial related terminology in chiropractic program courses, chiropractic accreditation standards, and chiropractic examining board testing content in the United States. Chiropr Man Ther.

[22] Cameron A. Registrant Survey 2020. General Chiropractic Council; 2021.

[23] AECC University College. https://www.aecc.ac.uk/clinics/clinical-services/student-chiropractic-clinic/; 2023.

[24] Willig C. EBOOK: introducing qualitative research in psychology. McGraw-hill education (UK); 2013.

[25] Willig C. Perspectives on the epistemological bases for qualitative research. 2012.

[26] Bhaskar R. A realist theory of science. Routledge; 2013.

[27] Wiltshire G, Ronkainen N (2021). A realist approach to thematic analysis: making sense of qualitative data through experiential, inferential and dispositional themes. J Crit Realism.

[28] Clarke V, Braun V. Thematic analysis: a practical guide. Thematic Anal. 2021:1–100.

[29] Braun V, Clarke V (2021). To saturate or not to saturate? Questioning data saturation as a useful concept for thematic analysis and sample-size rationales. Qualitative Res Sport Exerc Health.

[30] Malterud K, Siersma VD, Guassora AD (2016). Sample size in qualitative interview studies: guided by Information Power. Qual Health Res.

[31] Microsoft. Microsoft Teams software (version 1.5.00.28361). 2022.

[32] Clarke V, Braun V, Hayfield N. Thematic analysis. Qualitative psychology: A practical guide to research methods. 2015;3:222– 48.

[33] 12) NV. QSR International Pty Ltd.; 2018.

[34] Braun V, Clarke V (2019). Reflecting on reflexive thematic analysis. Qualitative Res Sport Exerc Health.

[35] Bandura A, Walters RH. Social learning theory. Englewood cliffs Prentice Hall; 1977.

[36] Bolton J (2000). Trust and the healing encounter: an examination of an unorthodox healing performance. Theor Med Bioeth.

[37] Bishop FL, Massey Y, Yardley L, Lewith GT (2011). How patients choose acupuncturists: a mixed-methods project. J Altern Complement Med.

[38] Kleiner MJ, Kinsella EA, Miciak M, Teachman G, McCabe E, Walton DM (2023). An integrative review of the qualities of a ‘good’physiotherapist. Physiother Theory Pract.

[39] Unsgaard-Tøndel M, Søderstrøm S. Therapeutic Alliance: Patients’ Expectations Before and Experiences After Physical Therapy for Low Back Pain-A Qualitative Study With 6-Month Follow-Up. Phys Ther. 2021;101(11). 10.1093/ptj/pzab187 PMC8632789 34339506

[40] Moore AJ, Holden MA, Foster NE, Jinks C (2020). Therapeutic alliance facilitates adherence to physiotherapy-led exercise and physical activity for older adults with knee pain: a longitudinal qualitative study. J Physiotherapy.

[41] Fuertes JN, Mislowack A, Bennett J, Paul L, Gilbert TC, Fontan G (2007). The physician-patient working alliance. Patient Educ Couns.

