# Supplementary material for: The development of working alliance in early stages of care from the perspective of patients attending a chiropractic teaching clinic
Source: Chiropr Man Therap. 2024 Mar 21;32:10. doi: 10.1186/s12998-023-00527-8 (PMC10958961; doi:10.1186/s12998-023-00527-8)
Supplement: Supplementary file 1 — Supplementary Material 1: Topic Guide [file 12998_2023_527_MOESM1_ESM.docx]

**Supplementary File 1 - Topic Guide**

| **Theme** | **Questions** |
| --- | --- |
| Introduction | Thank you for agreeing to take part in this interview. It will last around 45 minutes. Please feel free to pause or stop the interview at any point. As you know, the conversation will be recorded, but your personal information will remain confidential. The aim of the interview is to understand how the relationship between yourself, and your chiropractor develops. This includes how you both relate, make decisions, and work together to achieve the goals of care.   - Do you have any questions before we begin? |
| General Working Alliance and Expectations | - What did you think your chiropractor would be like before you met them? - And now you have met them, what were your first impressions? - What qualities do you think are important for a chiropractor? [personal characteristics, professional characteristics/skills] - What comes to mind when you think about the relationship between you and the chiropractor? - In your opinion, what would be the role of this relationship during your treatment? - What do you think is important for this relationship? |
| Agreement and Collaboration | - What do you think your role is during the chiropractic treatment? - What do you want to get out of coming for chiropractic? - What do you think your chiropractor wants to achieve? - What was the role of goal setting during your first appointment? - To what extent were you involved in making decisions during the appointment? - What do you think would influence this decision-making process? |
| Patient-Centredness / Individualised Care | - What do you expect from your chiropractor to consider when developing your treatment plan? - In your opinion, what factors in your life would influence your treatment progress? - How can your chiropractor take them into account? |
| Communication | - What would help you and your chiropractor to communicate effectively? - How can your chiropractor make you feel heard and understood? - What could cause tension between you and your chiropractor? |
| Bond | - What can the chiropractor do to gain your trust? - What do you view as important for the professional bond between you and chiropractor? |
| Personal and Professional Characteristics | - Which of the chiropractor’s personal characteristics do you think would influence your relationship? - Which of the chiropractor’s professional skills do you think would influence your relationship? - Which of your personal characteristics do you think would influence the relationship? |
