# Supplementary material for: The development of working alliance in early stages of care from the perspective of patients attending a chiropractic teaching clinic
Source: Chiropr Man Therap. 2024 Mar 21;32:10. doi: 10.1186/s12998-023-00527-8 (PMC10958961; doi:10.1186/s12998-023-00527-8)
Supplement: Supplementary file 2 — Supplementary Material 2: Checklist [file 12998_2023_527_MOESM2_ESM.docx]

**Chiropractic & Manual Therapies MANUSCRIPT REVISION CHECKLIST**

| **Manuscript title:** | The Development of Working Alliance in Early Stages of Care from the Perspective of Patients Attending a Chiropractic Teaching Clinic |
| --- | --- |
| **First author name:** | Dima Ivanova |

***Upload this completed checklist*** with your manuscript in the editorial submission system. If an item is not relevant write “N/A”.

| **ITEM** | **Mark with X, or provide number** |
| --- | --- |
| **Enter total word count** | N=10230 |
| **Enter Abstract word count (maximum 350 words)** | N=332 |
| **Abstract**   - Full structured abstract required for research articles and systematic reviews - Ensure the objectives and the results in the abstract correspond with those in the main text - Ensure the abstract in the main manuscript is the same as the abstract in the submission system | x |
| **Article Processing Charges (APCs):** if your manuscript meets the criteria, apply for APC coverage by the journal partners. See here for details: <https://chiromt.biomedcentral.com/criteria>  **You need to apply for this APC coverage *before* submitting your manuscript.** | x |
| **Reporting guidelines:** Research manuscripts must conform to the appropriate reporting guidelines, where relevant. Authors should refer to the [EQUATOR](http://www.equator-network.org/) network website for further information on the available reporting guidelines for health research. Checklists are available for a number of study designs, including the following designs commonly submitted to CMT: randomized controlled trials ([CONSORT](http://www.consort-statement.org/)) and protocols ([SPIRIT](http://www.spirit-statement.org/)); systematic reviews ([PRISMA](http://www.prisma-statement.org/)); observational studies ([STROBE](http://www.strobe-statement.org/)); qualitative studies ([COREQ](http://www.equator-network.org/reporting-guidelines/coreq/)); and case reports ([CARE](http://www.care-statement.org/)). Note this list is not exhaustive so refer to the [EQUATOR](http://www.equator-network.org/) website for other guidelines. | *Insert name of relevant reporting guideline:*  COREQ |
| If a **checklist** exists for the relevant reporting guideline for your manuscript, upload this with your submission. | **x** |
| **Ethics approval:** include name of ethics committee(s) or institutional review board(s) and **the approval number**. For further details of the journal’s editorial policies and ethical guidelines see:  <https://www.biomedcentral.com/getpublished/editorial-policies#ethics+and+consent> | x |
| **Date of study/data collection:** must be included in **abstract and main manuscript text** | x |
| **Statistical methods:** must be described in the methods section | x |
| **Numbers and percentages:**   - Provide actual numbers (with denominators) in the text and tables, in addition to percentages if used (do not use percentages if the denominator is <100) | x |
| - Confirm that all numbers and percentages in the article, especially in the Abstract, Results, Tables and Figures are accurate and consistent, and in the Tables, that the column totals are correct. | x |
| **Patient consent:** For all manuscripts that include details, images, or videos relating to individual participants, written informed consent for the publication of these must be obtained from the participants (or their parent or legal guardian in the case of children under 16) and a statement to this effect should appear in the manuscript. | x |
| **References:**   - Must be in BioMed Central style, within text citations should be in square brackets and all references checked for accuracy. See: <https://chiromt.biomedcentral.com/submission-guidelines/preparing-your-manuscript> | x |
| - Ensure you cite the primary reference to support your statements, for example cite a primary research article rather than a narrative review to support your assertions | x |
| - Internet references must include full details, not just the URL, and the URL must be that of the final destination, not just the home page. State the date you last accessed the URL | x |
| **Permission to reproduce items:** if any material has been previously published elsewhere, **permission must be obtained from the copyright owner** to reproduce the item in *Chiropractic & Manual Therapies*. Further, if you do not own copyright of any image, text or other material in your manuscript you MUST provide evidence of permission for publication under the creative commons licence, and attribute appropriately in the manuscript. | n/a |
| **Acknowledgements and “personal communications”:** Authors require written permission from all those mentioned in the Acknowledgements section. Keep this on file in case the editors request evidence of this. | x |
| **Competing interests:** Authors must disclose any financial and non-financial competing interests. Think of it in this way: you should disclose any competing interests that may cause you embarrassment were they to become public after the publication of the manuscript. See: <http://www.biomedcentral.com/getpublished/editorial-policies#competing+interests> | x |
| **Copyediting service:** Non-native speakers of English may choose to make use of a copyediting service, or the editors may recommend this if there are language issues with the paper. See here under “Style and language”: <https://chiromt.biomedcentral.com/submission-guidelines/preparing-your-manuscript> | x |
| **PROOF READ** the manuscript carefully and make any final changes. Chiropractic & Manual Therapies will NOT copyedit submitted manuscripts for style or language. Authors are advised to write clearly and simply, and to have their article checked by colleagues before final submission. | x |
| **ALL authors must approve the submitted version of the manuscript** | x |
